# Agreement between glandular ultrasonography and histopathology of minor salivary glands in adults with sicca syndrome

**DOI:** 10.1007/s10067-025-07650-2

**Published:** 2025-09-15

**Authors:** José S. Cortés, Wilder Carvajal, Alejandro Correa, Karen Veloza, Eybar Díaz, Luis J. Cajas

**Affiliations:** 1 Especialidad en Reumatología, Facultad de Medicina, Universidad Nacional de Colombia, Bogotá, 111321 Colombia; 2 Departamento de Reumatología, Hospital Universitario Nacional de Colombia, 111321 Bogotá, Colombia; 3 Hospital Central de la Policía Nacional, Bogotá, 111321 Colombia; 4 Asociación Colombiana de Reumatología - ASOREUMA, Bogotá, Colombia

**Keywords:** Histology, Lacrimal glands, Salivary glands, Sjogren’s disease, Ultrasonography

## Abstract

**Introduction/Objectives:**

Sjögren’s disease is an autoimmune disease that primarily affects the exocrine glands. Agreement between glandular ultrasound and focus score has been described as moderate to strong. The objective of this study was to assess the agreement between ultrasound findings of salivary and lacrimal glands and the histopathological results of minor salivary gland biopsies in patients with sicca syndrome seen at a quaternary care hospital in Colombia.

**Methods:**

This was an ambispective cohort study. A total of 66 patients were included, of whom 42 were classified as having Sjögren’s disease and 24 as having non-Sjögren sicca syndrome, according to the 2016 ACR/EULAR classification criteria. Focus scores were obtained from minor salivary gland biopsy reports, and ultrasound of the lacrimal and salivary glands was performed.

**Results:**

Ultrasound findings showed that 57.6% of patients presented glandular abnormalities, most frequently in the submandibular glands. The agreement between ultrasound findings and minor salivary gland biopsy was low (agreement rate: 57%, Kappa: 0.178). Advanced age (> 68 years) was identified as a factor negatively influencing the agreement. Parotid gland ultrasound had high specificity (100%) for the diagnosis of Sjögren’s disease, but limited sensitivity (24–30%). No significant association was found between abnormalities in lacrimal glands and the diagnosis of Sjögren’s disease.

**Conclusion:**

Although glandular ultrasound may serve as a complementary tool, it cannot yet replace minor salivary gland biopsy for the diagnosis of Sjögren’s disease. Further studies are needed to confirm its diagnostic performance and agreement with salivary gland histopathology.

**Table:** 

**Key Points** • *Overall agreement between ultrasound and minor salivary gland biopsy was low in this cohort* • *Older age (>68 years) was associated with reduced agreement between imaging and histopathology* • *Parotid gland ultrasound showed high specificity but limited sensitivity for classifying Sjögren’s disease*

**Supplementary Information:**

The online version contains supplementary material available at 10.1007/s10067-025-07650-2.

## Introduction

*Sicca* syndrome refers to the presence of dry eyes or dry mouth [1]. Sjögren’s disease (SjD) is a chronic, systemic, autoimmune disease that primarily affects the exocrine glands [1, 2]. SjD affects 0.01–0.72% of the global population [3, 4]. In Colombia, a prevalence between 0.12% and 0.5% has been documented, with higher rates among individuals aged 65–69 years [4, 5]. The female-to-male ratio in Colombia is approximately 4.6:1 [5]. Dryness, pain, and fatigue are considered hallmark symptoms of this condition [6]. Diagnosis is based on clinical, serological, and histopathological criteria; some imaging findings may also be helpful [3]. No single criterion is sufficient to diagnose SjD. Nevertheless, minor salivary gland histopathology remains a key diagnostic component. The characteristic histological finding is focal lymphocytic sialadenitis in labial salivary gland biopsies [7]. It has been reported that up to 10% of patients undergoing minor salivary gland biopsy (MSGB) may experience complications, the most common being sensory alteration at the incision site [7].

Sound is a mechanical energy transmitted through pressure waves in a medium [8]. Ultrasound is based on this principle, producing images via a transducer that emits and receives sound signals in media such as air, blood, or soft tissue [9]. Salivary gland ultrasound has developed into a diagnostic technique with good performance, safety, relative affordability, and broad availability. In 2019, the Outcome Measures in Rheumatology (OMERACT) group established definitions for normal and abnormal salivary gland findings in SjD. Greyscale imaging was proposed, with descriptions of hypoechoic/anechoic areas and fatty or fibrotic lesions [10]. A classification system was developed, including a semiquantitative four-grade scale and a qualitative two-grade scale [11]. It has been suggested that in patients with negative anti-Ro antibodies and a normal ultrasound (grade 0–2), SjD is unlikely and MSGB may be avoided. When ultrasound is abnormal, it has a specificity of 91% for SjD diagnosis [12].

In medical research, observer agreement analysis can provide information on the reliability of a scoring system [13]. Observers may be physicians classifying patients or diagnostic tools used to evaluate disease presence [13]. The appropriate measure of agreement depends on the variable type—continuous, ordinal, or nominal categorical [13]. Early methods focused on the proportion of agreement, but this approach did not account for agreement due to chance [13]. Cohen proposed the kappa coefficient as a correction for chance agreement [13, 14]. Cohen’s kappa is widely used to assess agreement between two raters or tools with nominal categorical variables [15].

This study aimed to evaluate the agreement between ultrasound findings of the submandibular, parotid, and lacrimal glands and the histopathological results of MSGBs, as well as to explore the diagnostic performance of ultrasound for SjD in adult patients with *sicca* syndrome seen at a quaternary care rheumatology outpatient clinic in Colombia.

## Methods

### Study design

This was a quantitative, observational, analytical, ambispective cohort study—retrospective for data collection from clinical records and prospective for the performance of ultrasound assessments and the inclusion of newly diagnosed *sicca* patients without previous clinical documentation.

### Patients

The study was approved by the local Bioethics and Research Committee under act CEI-HUN-ACTA-2024–08. A non-probabilistic convenience sampling strategy was employed. Adult patients (> 18 years) with a diagnosis of *sicca* syndrome attending the rheumatology outpatient clinic were invited to participate if they had either a completed or pending report of MSGB. Those who agreed to participate provided written informed consent after receiving an explanation of the study and clarification of any questions. Based on previous reports showing a kappa coefficient of 0.82 [16], for the agreement between salivary or lacrimal gland ultrasound and MSGB in patients with *sicca* symptoms, a sample size of at least 37 patients was calculated, assuming a 90% statistical power and a 50% event proportion, with an estimated 10% data loss. Patient recruitment lasted six months. At the end of the inclusion period, patients were classified as having SjD if they fulfilled the 2016 ACR/EULAR classification criteria [17], or as having non-Sjögren *sicca* syndrome otherwise. They were also classified as having agreement or disagreement between ultrasound and MSGB findings.

Patients were excluded if they met any of the following criteria: history of surgical resection of salivary or lacrimal glands; history of trauma to these areas; history of head and neck radiotherapy; positive serology for hepatitis C virus or HIV; diagnosis of sarcoidosis, amyloidosis, or IgG4-related disease; MSGB pathology report older than 12 months; or failed MSGB result.

### Clinical data

Data was extracted from patients' clinical records at the referral hospital. Sociodemographic variables such as age and sex were collected; sex was recorded as the binary sex assigned at birth. Clinical variables included weight, height, duration of *sicca* symptoms, and presence of ocular, oral, or genital dryness. Additional data included Schirmer’s test results and medical history.

Laboratory parameters extracted from the clinical records included: complete blood count (leukocytes, neutrophils, lymphocytes, platelets/mm^3^), C3 and C4 complement, C-reactive protein (CRP), erythrocyte sedimentation rate (ESR), rheumatoid factor (RF), anti-cyclic citrullinated peptide (anti-CCP) antibodies, antinuclear antibody (ANA) patterns and titres, extractable nuclear antigen (ENA) antibodies (Ro, La, RNP, Sm), and others. When multiple results were available, the most recent result closest in time to both the MSGB and the ultrasound was selected.

### Salivary and lacrimal gland ultrasound

Participants who provided consent were referred to a designated ultrasound room in the hospital where the study was conducted.. Ultrasound was performed using an Esaote MyLab Omega machine with a linear transducer (Esaote IH 6–18 MHz). Patients were positioned supine with the neck hyperextended and the head rotated contralaterally, as previously recommended [18]. Longitudinal views were obtained of the bilateral parotid and submandibular salivary glands and lacrimal glands. Ultrasound images were interpreted using the OMERACT classification, which includes a four-grade semiquantitative scale and a two-grade qualitative scale [11]. All ultrasound images were acquired and scored by a rheumatologist who holds a master’s degree in rheumatologic musculoskeletal ultrasound and has more than five years of experience, including expertise in salivary and lacrimal gland ultrasound., blinded to the clinical and histopathological data. A semiquantitative score ≥ 2 was considered abnormal [11]. Scoring was supported using a reference atlas. For analysis, findings were stratified by individual gland and grouped by region (submandibular glands, parotid glands, salivary glands, lacrimal glands, and all glands combined).

#### Statistical analysis

Data were entered into a REDCap database and analysed using Stata 17®. Normality was assessed using the Shapiro–Wilk test. Continuous variables are presented as median (minimum-maximum), and categorical variables as frequencies and percentages. Between-group differences for continuous variables were assessed using the Mann–Whitney U test (for non-normal distributions) or Student’s t-test (for normal distributions). ANOVA or Kruskal–Wallis tests were used for comparisons involving more than two groups. Categorical variables were analysed using the chi-square test or Fisher’s exact test, as appropriate. Where relevant, odds ratios (ORs) were calculated as measures of association. Agreement between MSGB histopathology and ultrasound findings was evaluated using Cohen’s Kappa coefficient, stratified by gland group. Receiver Operating Characteristic (ROC) curve analysis was used to identify the optimal cut-off for the number of abnormal glands to differentiate SjD from non-SjD. The area under the ROC curve (AUC) and its confidence interval (CI) were reported. Variables with > 30% missing data were excluded from inferential analyses. A p-value < 0.05 was considered statistically significant.

## Results

**Fig. 1:**
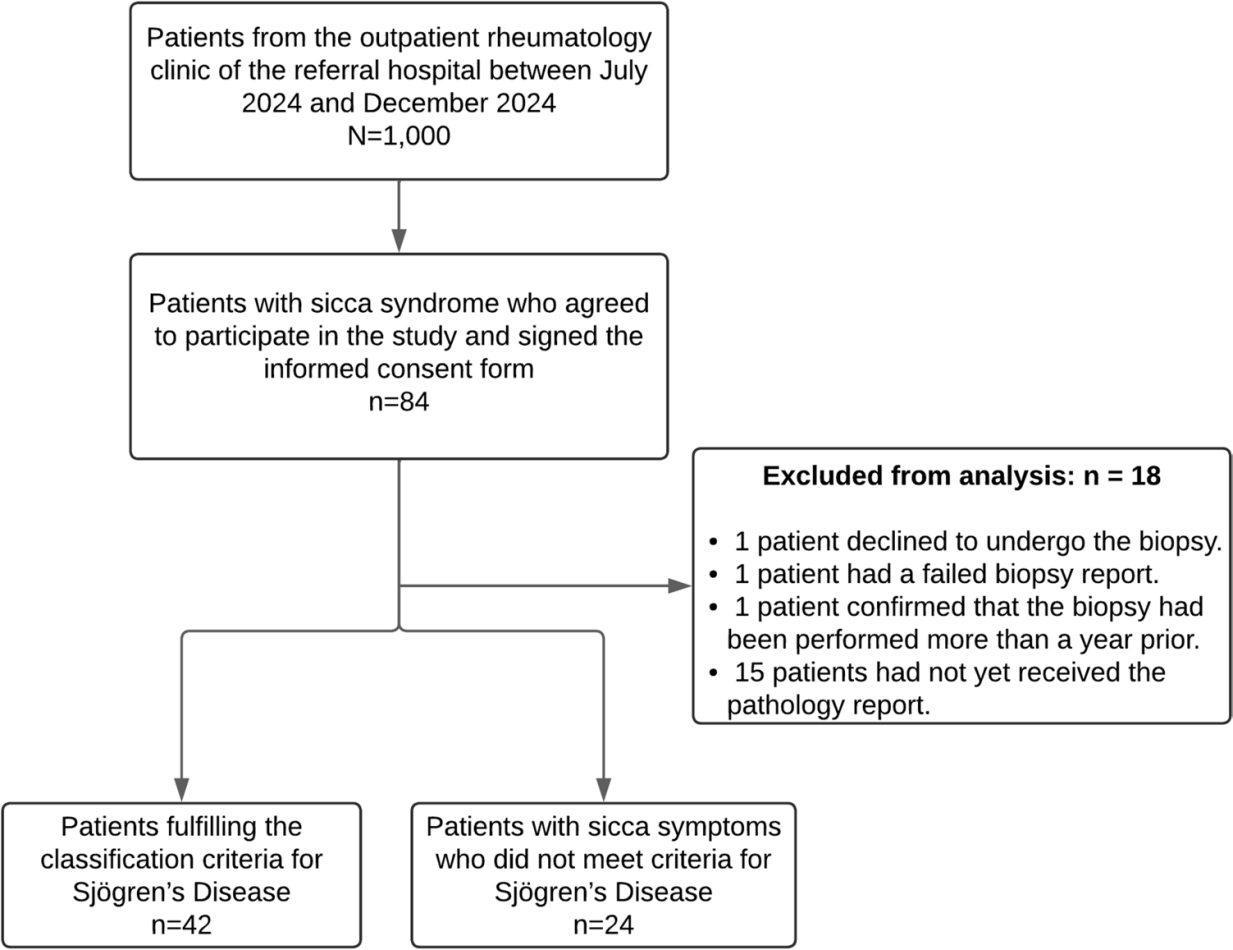
Study flowchart

From July to December 2024, patients attending the Rheumatology outpatient clinic of the referral hospital were invited to participate in the study (*N* = 1,000). During the recruitment period, 84 patients with *sicca* syndrome agreed to participate and signed informed consent forms. Of these, 18 patients were excluded from the analysis, mainly due to the unavailability of MSGB reports. The final analysis included 66 patients, of whom 42 were classified as having SjD and 24 as having non-Sjögren *sicca* syndrome (Fig. 1). Out of the 66 patients, 38 (57%) showed agreement between glandular ultrasound and MSGB.

### Sociodemographic characteristics and comorbidities

Overall, 92% of the patients were female, and 63% were classified as having SjD. Approximately 15% of the patients had at least one comorbidity. Table 1 and Supplementary Table S1 present the sociodemographic and comorbidity characteristics of the participants.

**Table 1:** Sociodemographic characteristics and comorbidities of patients with sicca syndrome classified as Sjögren’s and non-Sjögren’s disease

Sociodemographic variables	All, *n* = 66		SjD, *n* = 42		no SjD, *n* = 24		*p*
Sex, Female^a^	61	92.4	40	95.2	21	87.50	0.345
Age, years^b^	62	29-82	63	29-82	59	32-71	0.076
Weight, Kg^b^	63	47-150	62	47-150	67.5	49-97	0.270
Height, cm^b^	156	60-179	155	60-170	157	144-179	0.436
BMI,Kg/m^2^^b^	25.8	18.4-41.7	25.1	18.4-41.7	26.4	19.9-32.8	0.381
Comorbidities^a^							
Rheumatoid arthritis	6	9.1	3	7.1	3	12.5	0.66
Type 2 diabetes mellitus	13	19.7	8	19.0	5	20.8	>0.999
Systemic sclerosis	2	3.0	1	2.4	1	4.2	>0.999
Interstitial lung disease	9	13.6	4	9.5	5	20.8	0.268
Fibromyalgia	11	16.7	7	16.7	4	16.7	>0.999
Hypertension	19	28.8	12	28.6	7	29.2	>0.999
Hypothyroidism	13	19.7	10	23.8	3	12.5	0.345
Systemic lupus erythematosus	2	3.0	1	2.4	1	4.2	>0.999
Osteoarthritis	13	19.7	8	19.0	5	20.8	>0.999
Osteoporosis	13	19.7	8	19.0	5	20.8	>0.999

^a^ Values expressed as frequency (percentage). *p*-values correspond to chi-square or Fisher’s exact test

^b^ Values expressed as median (minimum–maximum). SjD: Sjögren’s disease. *p*-values correspond to Mann–Whitney U test. Comparisons were made between SjD and non- SjD *sicca* groups

No significant differences were found between the SjD and non-SjD groups in terms of sociodemographic variables or comorbidities. One case was identified for each of the following conditions: ANCA-associated vasculitis, antiphospholipid syndrome, spondyloarthropathies, uveitis, autoimmune hepatitis, idiopathic inflammatory myopathy, central nervous system vasculitis, dementia, skin cancer, and non-alcoholic fatty liver disease. Only 15% of patients had been evaluated by ophthalmology for dry eye assessment. Of the 66 patients, 38 (57%) demonstrated concordance between glandular ultrasound and MSGB (Supplementary Table S1). Patients without agreement were found to be older.

### Clinical and laboratory characteristics

Table 2 and Supplementary Table S2 display the laboratory findings by patient group. Comparison between the SjD and non-SjD groups showed statistically significant differences in anti-Ro antibody positivity, focus scores, and presence of RF. Seven patients (10%) had a focus score ≥ 1.

**Table 2:** Clinical and laboratory characteristics of patients with sicca syndrome classified as Sjögren’s and non-Sjögren’s disease

Variables	All, *n* = 66		SjD, *n* = 42		no SjD, *n* = 24		*p*
Years with *sicca* symptoms^b^	3	0-30	3	0-30	2.5	1-30	0.901
Dry eyes symptom^a^	55	83.3	36	85.7	19	79.2	0.511
Abnormal Schirmer test^a,c^	32	78.0	21	50.0	11	45.8	0.177
Dry mouth symptom^a^	55	83.3	34	81.0	21	87.5	0.733
Genital dryness symptom^a^	31	47.0	18	42.9	13	54.2	0.446
Anti-Ro titres, U/L^b^	4.5	4.5-640	4.5	28.5-640	4.5	4.5	<0.001
Positive anti-Ro^a^	24	36.4	24	57.1	0	0.0	<0.001
Focus score^b^	0.58	0-10.6	1.11	0-10.6	0	0-0.8	<0.001
Focus score ≥1^a^	32	48.5	32	76.2	0	0.0	<0.001
Rheumatoid factor^a^	13	19.7	12	28.6	1	4.2	0.023
Anti-cyclic citrullinated peptides^a^	5	7.6	3	7.1	2	8.3	>0.999
Antinuclear antibodies^a^	45	68.2	33	78.6	12	50.0	0.027
C3, mg/dL^b^	123	84-155	123	84-155	123	99-148	0.450
C4, mg/dL^b^	23.5	12-39	20	12-39	26	13-32	0.466
C-reactive protein, mg/dL^b^	0.38	0-4.53	0.39	0-4.53	0.35	0.08-0.68	0.636
ESR, mm/h^b^	15	1-28	15	1-38	14.5	5-28	0.942
Leukopenia^a^	7	10.6	6	14.3	1	4.2	0.408
Lymphopenia^a^	2	3.0	2	4.8	0	0.0	0.530

^a^ Values expressed as frequency (percentage). *p*-values correspond to chi-square or Fisher’s exact test

^b^ Values expressed as median (minimum–maximum). *SjD*: Sjögren’s Disease. *ESR:* erythrocyte sedimentation rate. *p*-values correspond to Mann–Whitney U test. Comparisons were made between SjD and non- SjD *sicca* groups

^c^ Percentage of total number of patients with results of Schirmer test, *n*=41

The most common ANA pattern was AC-4 (*n* = 35.53%). Three patients had reports showing two ANA patterns: AC-4 + AC-22, AC-4 + AC-16, and AC-1 + AC-3. One patient was positive for anti-Sm, two for anti-RNP, five for anti-La, and one for anti-Scl-70. Seven patients had reports for anti-MPO and anti-PR3 antibodies, of which two were positive for each. Five patients had reports of p-ANCAs and c-ANCAs, with only one patient testing positive for p-ANCAs. Fourteen patients had antiphospholipid antibody profiles; two were positive for lupus anticoagulant, anti-cardiolipin IgG/IgM, and anti-β2-glycoprotein I IgG/IgM. Two patients were positive for anti-smooth muscle antibodies, and one for anti-mitochondrial antibodies. IgG subclass levels were measured in two patients and were within normal limits in both. No patients were positive for anti-dsDNA, anti-Jo1, HLA-B27, or cryoglobulins. Of 14 patients with serum protein electrophoresis, six had normal results (four of whom had a focus score > 1 but none > 4), and eight had a gamma peak, of which four had a focus score > 1 but < 4. No patients had low C3 levels; one had low C4 levels and a focus score of 4.

The Schirmer test was performed in 41 patients. No significant differences in comorbidities or laboratory parameters were found between patients with and without agreement between ultrasound and MSGB.

### Ultrasound of salivary and lacrimal glands

At least half of the patients underwent glandular ultrasound on the same day as the MSGB. The maximum time interval between MSGB and ultrasound was 8 months. Tables 3 and 4 summarise the ultrasound findings. The ultrasound-detected abnormalities did not exhibit a symmetrical distribution (Supplementary Data S2).

**Table 3:** Ultrasound of salivary and lacrimal glands with abnormal findings according to Sjögren’s disease classification, anti-Ro status, and focus score

Glands^a^	All, *n*=66		*Sicca* syndrome					Anti-Ro					Focus score				
			SjD, *n* = 42		no SjD, *n* = 24		*p*	Anti-Ro +, *n*=24		Anti-Ro -, *n*=42		*p*	≥1, *n*=32		<1, *n*=34		*p*
Right submandibular gland	26	39.4	23	54.8	3	12.5	0.00	14	58.3	12	28.6	0.01	17	53.1	9	26.5	0.02
Left submandibular gland	27	40.9	20	47.6	7	29.2	0.20	11	45.8	16	38.1	0.53	15	46.9	12	35.3	0.33
Subdmandibular glands	**32**	**48.5**	**25**	**59.5**	**7**	**29.2**	**0.02**	**15**	**62.5**	**17**	**40.5**	**0.08**	**18**	**56.3**	**14**	**41.2**	**0.13**
Right parotid gland	8	12.1	8	19.0	0	0	0.04	8	33.3	0	0.0	<0.005	4	12.5	4	11.8	0.92
Left parotid gland	8	12.1	8	19.0	0	0	0.04	7	29.2	1	2.4	0.001	4	12.5	4	11.8	0.92
Parotid glands	**10**	**15.2**	**10**	**23.8**	**0**	**0**	**0.01**	**9**	**37.5**	**1**	**2.4**	**<0.005**	**5**	**15.6**	**5**	**14.7**	**0.91**
Salivary glands	**32**	**48.5**	**25**	**59.5**	**7**	**29.2**	**0.02**	**15**	**62.5**	**17**	**40.5**	**0.08**	**18**	**56.3**	**14**	**41.2**	**0.13**
Right lacrimal gland	12	18.2	10	23.8	2	8.3	0.19	5	20.8	7	16.7	**0.67**	7	21.9	5	14.7	0.45
Left lacrimal gland	15	22.7	11	26.2	4	16.7	0.54	8	33.3	7	16.7	0.12	5	15.6	10	29.4	0.18
Lacrimal glands	**19**	**28.8**	**14**	**33.3**	**5**	**20.8**	**0.40**	**9**	**37.5**	**10**	**23.8**	**0.23**	**8**	**25.0**	**11**	**32.4**	**0.51**
At least one abnormal gland	**38**	**57.6**	**28**	**66.7**	**10**	**41.7**	**0.07**	**17**	**70.8**	**21**	**50.0**	**0.09**	**20**	**62.5**	**18**	**52.9**	**0.43**

^a^ Values expressed as frequency (percentage). *p*-values correspond to chi-square or Fisher’s exact test. *SjD*: Sjögren’s disease

**Table 4:** Distribution of abnormal glands in patients classified with and without Sjögren’s disease

Glands^a^	All, *n*=66		Sicca syndrome				
			SjD, *n* = 42		no SjD, *n* = 24		*p*
Number of abnormal salivary glands							
0	34	51.5	**17**	40.5	**17**	70.8	0.023
1	10	15.2	**6**	14.3	**4**	16.7	>0.999
2	13	19.7	**10**	23.8	**3**	12.5	0.345
3	3	4.5	**3**	7.1	**0**	0	0.295
4	6	9.1	**6**	14.3	**0**	0	0.079
Number of abnormal glands							
0	28	42.4	**14**	33.3	**14**	58.3	0.070
1	11	16.7	**7**	16.7	**4**	16.7	>0.999
2	13	19.7	**7**	16.7	**6**	25.0	0.523
3	3	4.5	**3**	7.1	**0**	0	0.295
4	6	9.1	**6**	14.3	**0**	0	0.079
5	4	6.1	**4**	9.5	**0**	0	0.288
6	1	1.5	**1**	2.4	**0**	0	>0.999

^a^ Values expressed as frequency (percentage). *p*-values correspond to chi-square or Fisher’s exact test. *SjD:* Sjögren’s disease

More than half of the patients (57.6%) had some degree of glandular abnormality, most commonly in the submandibular glands. No patient had an abnormal parotid gland with a normal submandibular gland. There was a statistically significant association between abnormalities in these two sets of glands (*p* < 0.05); 68.7% of patients with submandibular gland abnormalities had normal parotid glands (Supplementary Data 1A). A similar association was found between submandibular and lacrimal gland abnormalities (*p* = 0.03); 6 out of 34 patients (17.6%) with normal submandibular glands showed abnormalities in the lacrimal glands (Supplementary Data 1B). The association between parotid and lacrimal abnormalities was also significant (*p* = 0.002, Supplementary Data 1C). Parotid gland abnormalities were associated with anti-Ro positivity (*p* < 0.05), and submandibular (*p* = 0.02) and parotid gland (*p* = 0.01) abnormalities were associated with the final classification of SjD. No such association was found for lacrimal gland abnormalities.

There was no association between focus score > 1 and any abnormality in individual or grouped glands (Table 3), nor with the number of affected glands (*p* = 0.45) or the severity score (0 to 3) for submandibular (*p* = 0.55), parotid (*p* = 0.76), or lacrimal glands (*p* = 0.95). Similarly, there was no association between focus score > 1 and severity scores in any of the gland groups.

Lacrimal gland abnormalities were not associated with abnormal Schirmer test results (*p* = 0.17). Although 65% of patients with a positive Schirmer test had no lacrimal ultrasound abnormality, nearly all (91%) with positive test results had some form of glandular abnormality.

### Agreement

Table 5 shows the agreement between salivary gland ultrasound and MSGB. The overall agreement between ultrasound and MSGB was low. Among patients with a focus score ≥ 1, 37% had normal ultrasound results; conversely, 47% of those with negative biopsies showed abnormal findings in at least one gland. The agreement rate was 57%. Cohen’s kappa coefficients indicated poor agreement: submandibular glands: kappa = 0.178; parotid glands: kappa = 0.009; salivary glands: kappa = 0.178; lacrimal glands: kappa < 0; all glands combined: kappa = 0.095. When comparing patients with and without agreement, those without agreement were significantly older (p = 0.03). In an exploratory subanalysis excluding patients > 68 years old, agreement improved from poor to moderate (overall agreement: 65%, kappa = 0.316).

**Table 5:** Agreement of minor salivary gland biopsy and salivary gland ultrasound in patients classified with and without Sjögren’s disease

	MSGB positive, *n* = 32		MSGB negative, *n* = 34		Total
	SGUS-positive, *n* = 18	SGUS-negative, *n* = 14	SGUS-positive, *n* = 14	SGUS-negative, *n* = 20	
Sjögren’s disease	18	14	7	3	42
Not Sjögren’s disease	0	0	7	17	24

*MSGB:* minor salivary gland biopsy. *SGUS:* salivary glands ultrasound

### Diagnostic performance of salivary and lacrimal gland ultrasound

As an exploratory analysis, sensitivity and specificity were calculated for ultrasound findings in classifying SjD. Abnormal submandibular ultrasound had a sensitivity of 59% (CI: 44–73%) and specificity of 70% (CI: 50–85%). Abnormal parotid ultrasound had a sensitivity of 24% (CI: 13–38%) and a specificity of 100% (CI: 86–100%). These values were similar across anti-Ro positive and negative subgroups.

The performance of salivary gland ultrasound mirrored that of submandibular glands ultrasound, as all parotid abnormalities occurred in patients who also had submandibular gland abnormalities. No association was found between SjD classification and lacrimal gland ultrasound abnormalities, or between SjD and the combination of salivary and lacrimal gland findings.

An additional analysis of the optimal cut-off for the number of affected glands found that having two or three abnormal glands yielded sensitivities of 50% and 33%, and specificities of 75% and 100%, respectively (Supplementary Fig. 1). The ROC curve yielded an AUC of 69% (CI: 57–82%, *p *= 0.009). Moreover, having no abnormal salivary glands was associated with lower odds of SjD classification (OR = 0.28).

## Discussion

This is the first study in America that explores the agreement between ultrasound of the salivary and lacrimal glands and histopathological reports from MSGB in patients with *sicca* syndrome.

As previously reported, the female-to-male ratio among patients with SjD ranges from 4:1 to 15:1 [5]. In our study, no significant differences were observed between the SjD and non-SjD groups regarding clinical variables that could have confounded the interpretation of glandular ultrasound results. Only 15% of patients had been evaluated by ophthalmology for dry eye, raising concerns that patients may not be adequately referred for formal assessment—despite dry eye being one of the classification criteria for SjD [17]. Notably, Schirmer’s test, though part of the 2016 classification criteria, did not differ between SjD and non-SjD groups in our cohort. This finding warrants further investigation in larger samples.

In our study, we included patients with sicca syndrome and excluded only those with conditions listed as exclusion criteria for SjD in the ACR/EULAR classification criteria, to achieve a study population that more closely reflects the general population. Interestingly, no association was found between the presence of conditions such as type 2 diabetes mellitus or other autoimmune diseases and the agreement between SGUS and MSGB. This finding could be confirmed in future studies with greater representation of patients with other conditions associated with sicca syndrome and autoimmune diseases associated to SjD.

The interval between MSGB and glandular ultrasound was up to 8 months. Previous studies have shown that structural ultrasound abnormalities in patients with SjD or non-SjD *sicca* syndrome tend to remain stable over time. One cohort followed for a median of 23 months found that 80% of patients showed no change in ultrasound findings [19]. Similarly, the histological grade of inflammation in salivary gland biopsies has been reported to remain unchanged over a median follow-up of 55 months [20]. Furthermore, prior studies suggest that the use of reference atlases for ultrasound scoring can enhance observer performance [21]. This methodological approach was applied in the present study.

As previously reported [15, 21, 22, 23, 24, 25, 26, 27], the diagnostic performance of salivary gland ultrasound in SjD varies, with reported sensitivities from 55 to 90% and specificities from 67 to 98%. Agreement between ultrasound and MSGB has been reported at 83–91%, with Kappa values ranging from 0.48 to 0.82. Several studies have found significant associations between pathological ultrasound findings and anti-Ro52 positivity or hypergammaglobulinaemia, findings that were also replicated in our study. In our analysis, the diagnostic performance of ultrasound for SjD fell within the lower bounds of previously reported ranges. It is worth noting that some previous studies included patients with *sicca* symptoms regardless of underlying comorbidities [27], which may have increased diagnostic performance metrics.

Interestingly, we observed that parotid gland abnormalities had nearly 100% specificity for SjD, regardless of anti-Ro status, albeit with low sensitivity (20–30%). This suggests that in approximately one out of every 3 to 5 patients with *sicca* syndrome, the presence of parotid abnormalities could support the diagnosis of SjD and potentially obviate the need for MSGB. Similar performance was observed when using a threshold of three abnormal glands on ultrasound to differentiate SjD from non-SjD cases. Moreover, a normal glandular ultrasound was associated with lower odds of SjD classification (OR 0.2–0.3), underscoring the potential value of ultrasound as a rule-out tool. However, among anti-Ro negative patients with normal ultrasound findings, up to 30% would have been missing if MSGB had not been performed.

A particularly relevant finding was that patients without agreement between ultrasound and MSGB were significantly older. This raises the possibility that ultrasound performance varies across age groups. Ageing has been associated with nonspecific chronic sialadenitis, which may present ultrasound findings similar to those seen in SjD [28]. Given the frequency range of the transducer used in our study, it is plausible that glandular ultrasound captured changes not limited to focal lymphocytic sialadenitis but also those related to nonspecific inflammation. This may account for the agreement observed. In prior studies reporting better grades of agreement between ultrasound and MSGB [16, 26], the majority of patients were younger than 68 years, a threshold above which we found agreement to decrease. Future studies should explore the impact of age, transducer frequency, and patient stratification on ultrasound-histology agreement.

The study found a significant discrepancy in ultrasound echotexture abnormalities, with submandibular glands affected more frequently than parotid glands, with no parotid abnormalities occurring without submandibular involvement. This might be explained by anatomical differences, greater submandibular susceptibility to inflammatory changes, and age-related nonspecific sialadenitis, particularly in patients > 68 years, which reduces ultrasound-histopathology agreement. These findings align with prior studies [24, 25] reporting higher submandibular gland sensitivity for SjD, while parotid abnormalities show high specificity (100%) but low sensitivity (24–30%).

Although lacrimal gland ultrasound remains poorly standardised and highly subjective [18], we decided to explore its diagnostic potential. Our results showed no association between lacrimal abnormalities and SjD classification, anti-Ro positivity, or focus score > 1. Future studies may help clarify whether lacrimal ultrasound holds diagnostic value or could be used for disease monitoring.

We did not evaluate the relationship between SjD activity and SGUS using the OMERACT scale. Previous studies, such as a Danish single-centre study [29] using the OMERACT SGUS scoring system, found no significant correlation between SGUS severity and ESSDAI scores, suggesting that the degree of ultrasonographic glandular abnormality assessed by OMERACT does not necessarily reflect systemic disease activity as captured by ESSDAI in all cohorts.

Among the limitations of this study is the partially retrospective nature of clinical data collection. However, none of the key study variables showed significant data loss. Another potential limitation is the inclusion of pathology reports from multiple laboratories, which may have introduced variability in histological interpretation and focus score grading. In future studies, it may be relevant to include variables representing disease activity, such as ESSDAI or ESSPRI scores, as these could influence the degree of agreement between SGUS and MSGB. Incorporating these scores would allow for a better assessment of the relationship between ultrasound findings and clinical disease activity.Also future research could consider restricting analysis to reports from a single pathology lab to reduce heterogeneity. It would also be useful to design future studies that evaluate interobserver agreement in SGUS under similar conditions.

In conclusion, this study evaluated the agreement between ultrasound findings of salivary and lacrimal glands and minor salivary gland histopathology in patients with *sicca* syndrome. Although glandular ultrasound is a safe, accessible, and potentially useful tool, its agreement with biopsy was low to moderate, and it cannot currently replace histopathology. Parotid gland ultrasound demonstrated high specificity, suggesting its value as a complementary diagnostic tool, particularly in patients with multiple glandular abnormalities. Additionally, advanced age (> 68 years) was associated with reduced agreement, highlighting the importance of considering individual clinical factors in diagnostic interpretation. Based on these findings, further multicentre studies with more representative samples are recommended, as well as standardization of ultrasound technique and training, exploration of probe types, and evaluation of integrated diagnostic strategies using algorithms that combine clinical, serological, and imaging data. Multidisciplinary collaboration among rheumatology, ophthalmology, and pathology is essential to improve the diagnostic approach to *sicca* syndrome.

## Supplementary Information

Below is the link to the electronic supplementary material.

Supplementary file 1 (PNG 87.8 KB)

Supplementary file 2 (DOCX 48.3 KB)

## Data Availability

The datasets generated and analysed during the current study are available from the corresponding author upon reasonable request.
