# Supplementary figures and images for: Agreement between glandular ultrasonography and histopathology of minor salivary glands in adults with sicca syndrome
Source: Clin Rheumatol. 2025 Sep 15;44(11):4567–76. doi: 10.1007/s10067-025-07650-2 (PMC12568837; doi:10.1007/s10067-025-07650-2)

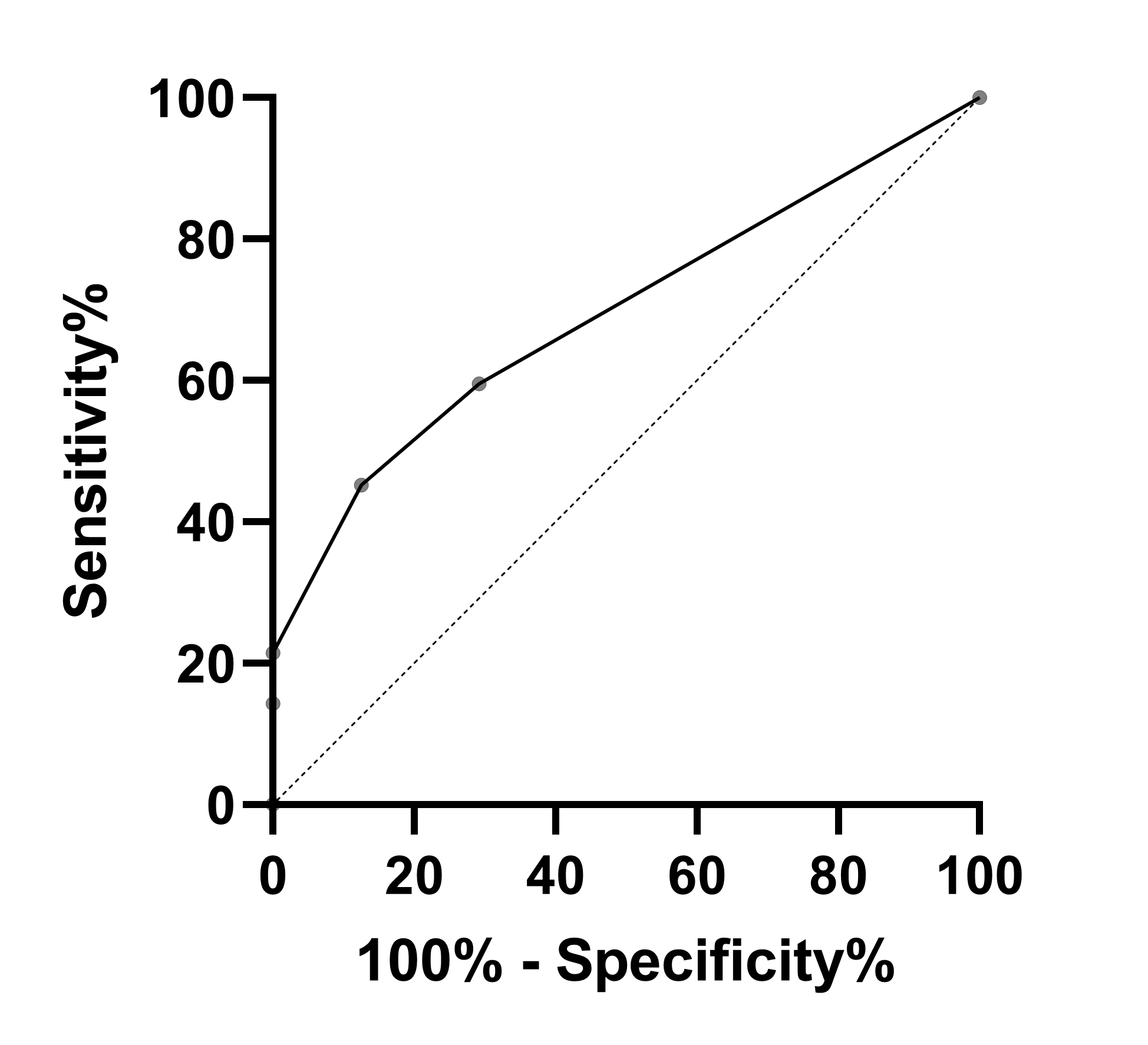

Supplement: Supplementary file 1 — (PNG 87.8 KB) [file 10067_2025_7650_MOESM1_ESM.png]
