# Supplementary material for: Agreement between glandular ultrasonography and histopathology of minor salivary glands in adults with sicca syndrome
Source: Clin Rheumatol. 2025 Sep 15;44(11):4567–76. doi: 10.1007/s10067-025-07650-2 (PMC12568837; doi:10.1007/s10067-025-07650-2)
Supplement: Supplementary file 2 — (DOCX 48.3 KB) [file 10067_2025_7650_MOESM2_ESM.docx]

**Clinical Rheumatology**

**Agreement between Glandular Ultrasonography and Histopathology of Minor Salivary Glands in Adults with Sicca Syndrome**

José S. Cortés^1^, Wilder Carvajal^1^, Alejandro Correa^1^, Karen Veloza^2,^ Eybar Díaz^3^, Luis J. Cajas^4^

^1^ Residente de Reumatología, Especialidad en Reumatología, Facultad de Medicina, Universidad Nacional de Colombia. Departamento de Reumatología, Hospital Universitario Nacional de Colombia. Bogotá, 111321, Colombia.

^2^ Médica, Hospital Central de la Policía Nacional, 111321, Colombia.

^3^ Especialidad en Reumatología, Facultad de Medicina, Universidad Nacional de Colombia. Departamento de Reumatología, Hospital Universitario Nacional de Colombia. Bogotá, 111321, Colombia.

^4^ Especialidad en Reumatología, Facultad de Medicina, Universidad Nacional de Colombia. Bogotá, 111321, Colombia.

Corresponding author:

José S. Cortés

Calle 44 #59-75, 111321, Teusaquillo, Bogotá, Cundinamarca

Email address: jocortesgu@unal.edu.co

| **Supplementary Table S1. Sociodemographic characteristics and comorbidities of the participants according to ultrasound–histological agreement** | | | | | | | |  |
| --- | --- | --- | --- | --- | --- | --- | --- | --- |
| **Sociodemographic variables** | **All, n = 66** | | **Agreement, n = 38** | | **No agreement, n = 28** | | **p** |  |
| Sex, Female^a^ | 61 | 92.4 | 35 | 92.1 | 21 | 75.00 | 0.083 |  |
| Age, years^b^ | 62 | 29-82 | 61 | 29-76 | 65 | 34-82 | 0.039 |  |
| Weight, Kg^b^ | 63 | 47-150 | 64 | 47-150 | 63 | 48-80 | 0.464 |  |
| Height, cm^b^ | 156 | 60-179 | 157 | 60-179 | 154 | 144-175 | 0.619 |  |
| BMI,Kg/m^2 b^ | 25.8 | 18.4-41.7 | 26.4 | 18.4-41.6 | 25 | 19.9-34.2 | 0.553 |  |
| **Comorbidities**^a^ |  |  |  |  |  |  |  |  |
| Rheumatoid arthritis | 6 | 9.1 | 4 | 10.5 | 2 | 7.14 | >0.999 |  |
| Type 2 diabetes mellitus | 13 | 19.7 | 8 | 21.1 | 5 | 17.86 | >0.999 |  |
| Systemic sclerosis | 2 | 3.0 | 1 | 2.6 | 1 | 3.57 | >0.999 |  |
| Interstitial lung disease | 9 | 13.6 | 5 | 13.2 | 4 | 14.29 | >0.999 |  |
| Fibromyalgia | 11 | 16.7 | 9 | 23.7 | 2 | 7.14 | 0.101 |  |
| Hypertension | 19 | 28.8 | 9 | 23.7 | 10 | 35.71 | 0.409 |  |
| Hypothyroidism | 13 | 19.7 | 8 | 21.1 | 5 | 17.86 | >0.999 |  |
| Systemic lupus erythematosus | 2 | 3.0 | 1 | 2.6 | 1 | 3.57 | >0.999 |  |
| Osteoarthritis | 13 | 19.7 | 6 | 15.8 | 7 | 25.00 | 0.369 |  |
| Osteoporosis | 13 | 19.7 | 7 | 18.4 | 6 | 21.43 | 0.765 |  |
| ^a^ Values expressed as frequency (percentage). p-values correspond to chi-square or Fisher’s exact test.  ^b^ Values expressed as median (minimum–maximum). p-values correspond to Mann–Whitney U test. Comparisons were made between agreement and no agreement groups. | | | | | | | |  |
|  |  |  |  |  |  |  |  |  |

| **Supplementary Table S2. Clinical and paraclinical characteristics of the participants according to ultrasound-histological agreement** | | | | | | | | | | |  |
| --- | --- | --- | --- | --- | --- | --- | --- | --- | --- | --- | --- |
| **Variables** | **All, n = 66** | | | **Agreement, n = 38** | | | **No agreement, n = 28** | | | **p** |  |
| Years with *sicca* symptoms^b^ | | 3 | 0-30 | | 3 | 0-30 | 2 | 0-20 | 0.25 | |  |
| Dry eyes symptom^a^ | | 55 | 83.3 | | 30 | 78.9 | 25 | 104.2 | 0.331 | |  |
| Abnormal Schirmer test^a,c^ | | 32 | 78.0 | | 17 | 44.7 | 15 | 62.5 | 0.619 | |  |
| Dry mouth symptom^a^ | | 55 | 83.3 | | 32 | 84.2 | 23 | 95.8 | >0.999 | |  |
| Genital dryness symptom^a^ | | 31 | 47.0 | | 17 | 44.7 | 14 | 58.3 | 0.804 | |  |
| Anti-Ro titres, U/L^b^ | | 4.5 | 4.5-640 | | 4.5 | 4.5-640 | 4.5 | 4.5-640 | 0.577 | |  |
| Positive anti-Ro^a^ | | 24 | 36.4 | | 12 | 31.6 | 12 | 50.0 | 0.439 | |  |
| Focus score^b^ | | 0.58 | 0-10.6 | | 0.58 | 0-10.6 | 0.65 | 0-6.4 | 0.812 | |  |
| Focus score ≥1^a^ | | 32 | 48.5 | | 18 | 47.4 | 14 | 58.3 | >0.999 | |  |
| Sjögren’s Disease^a^ | | 42 | 63.6 | | 22 | 57.9 | 20 | 83.3 | 0.307 | |  |
| Rheumatoid factor^a^ | | 13 | 19.7 | | 4 | 10.5 | 9 | 37.5 | 0.057 | |  |
| Anti-cyclic citrullinated peptides^a^ | | 5 | 7.6 | | 4 | 10.5 | 1 | 4.2 | 0.385 | |  |
| Antinuclear antibodies^a^ | | 45 | 68.2 | | 23 | 60.5 | 22 | 91.7 | 0.102 | |  |
| C-reactive protein, mg/dL^b^ | | 0.38 | 0-4.53 | | 0.38 | 0-1 | 0.37 | 0.04-4.53 | 0.809 | |  |
| C3, mg/dL^b^ | | 123 | 84-155 | | 123 | 86-155 | 124 | 84-148 | 0.638 | |  |
| C4, mg/dL^b^ | | 23.5 | 12-39 | | 20 | 12-31 | 27 | 13-39 | 0.145 | |  |
| ESR, mm/h^b^ | | 15 | 1-28 | | 15 | 2-36 | 11 | 1-38 | 0.944 | |  |
| Leukopenia^a^ | | 7 | 10.6 | | 5 | 13.2 | 2 | 8.3 | 0.689 | |  |
| Lymphopenia^a^ | | 2 | 3.0 | | 1 | 2.6 | 1 | 0.0 | >0.999 | |  |
| ^a^ Values expressed as frequency (percentage). p-values correspond to chi-square or Fisher’s exact test.  ^b^ Values expressed as median (minimum–maximum). p-values correspond to Mann–Whitney U test. ESR, erythrocyte sedimentation rate. Comparisons were made between agreement and no agreement groups.  ^c^ Percentage of total number of patients with results of Schirmer test, n=41. | | | | | | | | | | |  |
|  |  |  |  |  |  |  |  |  |  |  |  |
|  |  |  |  |  |  |  |  |  |  |  |  |

**Supplementary Data S1. Relationship between ultrasound abnormalities in submandibular, parotid, and lacrimal glands**

1. Submandibular and parotid gland abnormalities

|  | Normal parotid glands | 1 or 2 abnormal parotid glands | Total |
| --- | --- | --- | --- |
| Normal submandibular glands | 34 | 0 | 34 |
| 1 or 2 abnormal submandibular glands | 22 | 10 | 32 |
| Total | 56 | 10 | 66 |

1. Submandibular and lacrimal gland abnormalities

|  | Normal lacrimal glands - | 1 or 2 abnormal lacrimal glands | Total |
| --- | --- | --- | --- |
| Normal submandibular glands | 28 | 6 | 34 |
| 1 or 2 abnormal submandibular glands | 19 | 13 | 32 |
| Total | 47 | 19 | 66 |

1. Parotid and lacrimal gland abnormalities

|  | Normal lacrimal glands | 1 or 2 abnormal lacrimal glands | Total |
| --- | --- | --- | --- |
| Normal parotid glands | 44 | 12 | 56 |
| 1 or 2 abnormal parotid glands | 3 | 7 | 10 |
| Total | 47 | 19 | 66 |

**Supplementary Data S2. OMERACT classification of salivary and lacrimal glands using the semiquantitative 0–3 scale**

| **OMERACT score** | **Right submandibular gland** | **Left submandibular gland** | **Right parotid gland** | **Left parotid gland** | **Right lacrimal gland** | **Left lacrimal gland** |
| --- | --- | --- | --- | --- | --- | --- |
| **0** | 22 | 18 | 53 | 40 | 25 | 29 |
| **1** | 18 | 21 | 5 | 18 | 29 | 22 |
| **2** | 20 | 22 | 6 | 8 | 10 | 13 |
| **3** | 6 | 5 | 2 | 0 | 2 | 2 |

**Supplementary Figure S1. Receiver Operator Characteristics curve for the number of glands with ultrasound abnormalities to identify cases classified as Sjögren’s Disease**


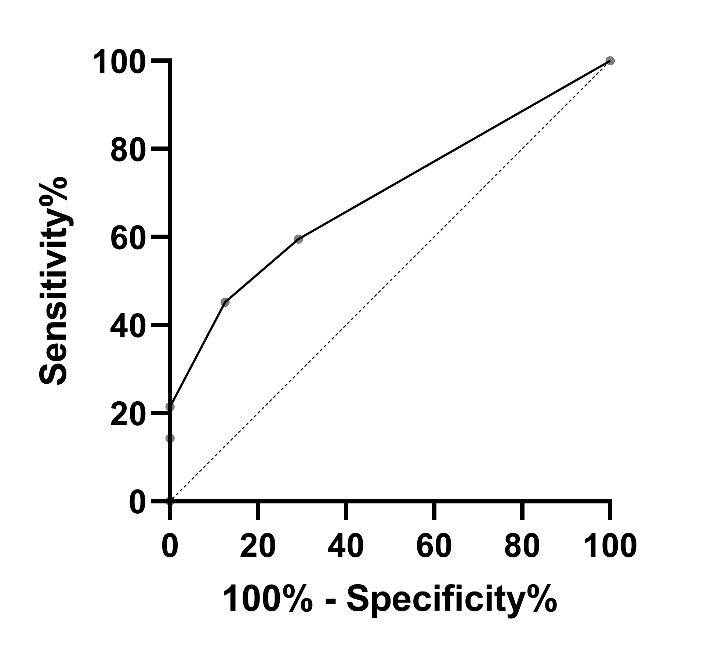
**.**
